# A systematic review and meta-analysis of the association of all types of beverages high in fructose with asthma in children and adolescents

**DOI:** 10.1186/s40795-024-00930-1

**Published:** 2024-09-18

**Authors:** Fatemeh Keshavarz, Mobina Zeinalabedini, Soraiya Ebrahimpour-Koujan, Leila Azadbakht

**Affiliations:** 1 Department of Community Nutrition, School of Nutritional Sciences and Dietetics, Tehran University of Medical Sciences, PO Box 1416643931, Tehran, Iran; 2 Students’ Scientific Research Center (SSRC), Tehran University of Medical Sciences (TUMS), Tehran, Iran; 3 Department of Clinical Nutrition, School of Nutritional Sciences and Dietetics, Shiraz University of Medical Sciences, Shiraz, Iran; 4 Diabetes Research Center, Endocrinology and Metabolism Clinical Sciences Institute, Tehran University of Medical Sciences, Tehran, Iran; 5 Department of Community Nutrition, School of Nutrition and Food Science, Isfahan University of Medical Science, Isfahan, Iran

**Keywords:** SSBs, Children, Asthma, Adolescents, High fructose corn syrup

## Abstract

**Background:**

Asthma has become the most common chronic condition among children in recent decades. Environmental factors, including food, drive its rise. Sweetened beverages are a staple of children’s diets and cause various health issues. Therefore, this research aims to evaluate the association of all types of high fructose beverages with asthma in children.

**Method:**

We assessed observational studies published before November 2023, obtained from PubMed, Scopus, and Web of Science. The quality of articles was assessed by using the Newcastle-Ottawa Scale. Studies with a pediatric population under 18 years that indicate the association between all kinds of beverages containing high fructose and asthma and evaluated risk estimates with 95% confidence intervals were included. We also followed Preferred Reporting Items for Systematic Reviews and Meta-analyses (PRISMA).

**Results:**

In the final analysis, we included eleven studies with 164,118 individuals. Twenty-one effect sizes indicated a significant positive association between sugar-sweetened beverages (SSBs) consumption and odds of asthma (OR: 1.28; 95% CI: 1.15–1.42; P_value_ < 0.001). Three effect sizes showed that total excess free fructose (tEFF) intake increases children’s asthma odds by 2.7 times (pooled OR: 2.73; 95% CI: 1.30–5.73; P_value_ =0.008). However, five effect sizes in 100% fruit juice failed to show statically association with asthma prevalence in children (pooled OR: 1.43; 95%CI: 0.91–2.23; P_value_ =0.12).

**Conclusion:**

In summary, SSB and tEFF raised asthma probabilities. No relationship was found between fruit juice and asthma in children and adolescents. We need more cohort studies with correct age selection to identify the precise link.

**Supplementary Information:**

The online version contains supplementary material available at 10.1186/s40795-024-00930-1.

## Introduction

Asthma is the most prevalent chronic disease in children characterized by inflammation in the upper airways. Asthma in children usually presents with wheezing, cough, and shortness of breath [1]. The prevalence of asthma in children has increased over the past few decades and made it an important public health concern worldwide [2, 3]. Childhood asthma often begins at an early age and up to 50% of people with asthma experience symptoms in the first 6 years of life [4]. Childhood asthma leads to many disabilities in children and causes difficulty with daily activities. Severe asthma in children causes lifelong outcomes like high risk of chronic obstructive pulmonary disease (COPD) and adulthood asthma [2].

Asthma results from a combination of genetic and environmental factors [5]. Childhood asthma risk factors include preterm asthma, prematurity, atopic dermatitis, obesity, early sensitization to food and aeroallergens, exposure to viral infections, and irritants such as tobacco smoke. However, environmental factors are primarily responsible for the worldwide increase in asthma initiation [6]. Accordingly, western lifestyle and dietary risk factors such as trans-fatty acids (TFA), animal products, fast foods, and sugar-sweetened beverages (SSBs) are associated with the risk of asthma [7, 8].

SBBs including fruit drinks and soda have become a major part of children’s diets which are an important source of added sugars and fructose [9]. SSBs cause obesity and increase the risk of chronic diseases such as type-2 diabetes mellitus, cardiovascular disease, some cancers, and asthma [10, 11]. Fructose, either as a part of sucrose or in the form of high fructose corn syrup (HFCS), is a common sweetener added to SSB [12]. Unabsorbed excess-free fructose in the intestinal tract produces glycation end products that trigger inflammation in the respiratory system [13, 14]. As SSBs emerge as a significant contributor to asthma, it becomes imperative to limit their consumption in children. Taking action in this regard is crucial for reducing the global burden of asthma.

Some epidemiological studies reported the association between sugar-sweetened beverages and childhood asthma, but the findings were inconsistent [7, 15, 16]. Some of these studies have assessed different types of beverages and they have been conducted on different age groups of children and adolescents. Only one meta-analysis assessed the association between soft drink consumption and asthma prevalence among both adults and children [7]. However, there is no meta-analysis on the association of various SSBs and beverages containing high fructose with asthma prevalence in children. Therefore, we performed this systematic review and meta-analysis to evaluate the relation between all types of beverages containing high fructose including; SSBs which are sweetened with HFCS or any excess free fructose, and 100% Fruit juice with odds of asthma in children and adolescents under 18 years old.

## Method

This study adhered to the Preferred Reporting Items for Systematic Review and Meta-Analysis (PRISMA) guidelines [17]. Full-text publications were double-screened by two independent reviewers throughout the selection process (FK and MZ). Any discrepancies were resolved by a third reviewer (LA).

### Search strategy

**Fig. 1:**
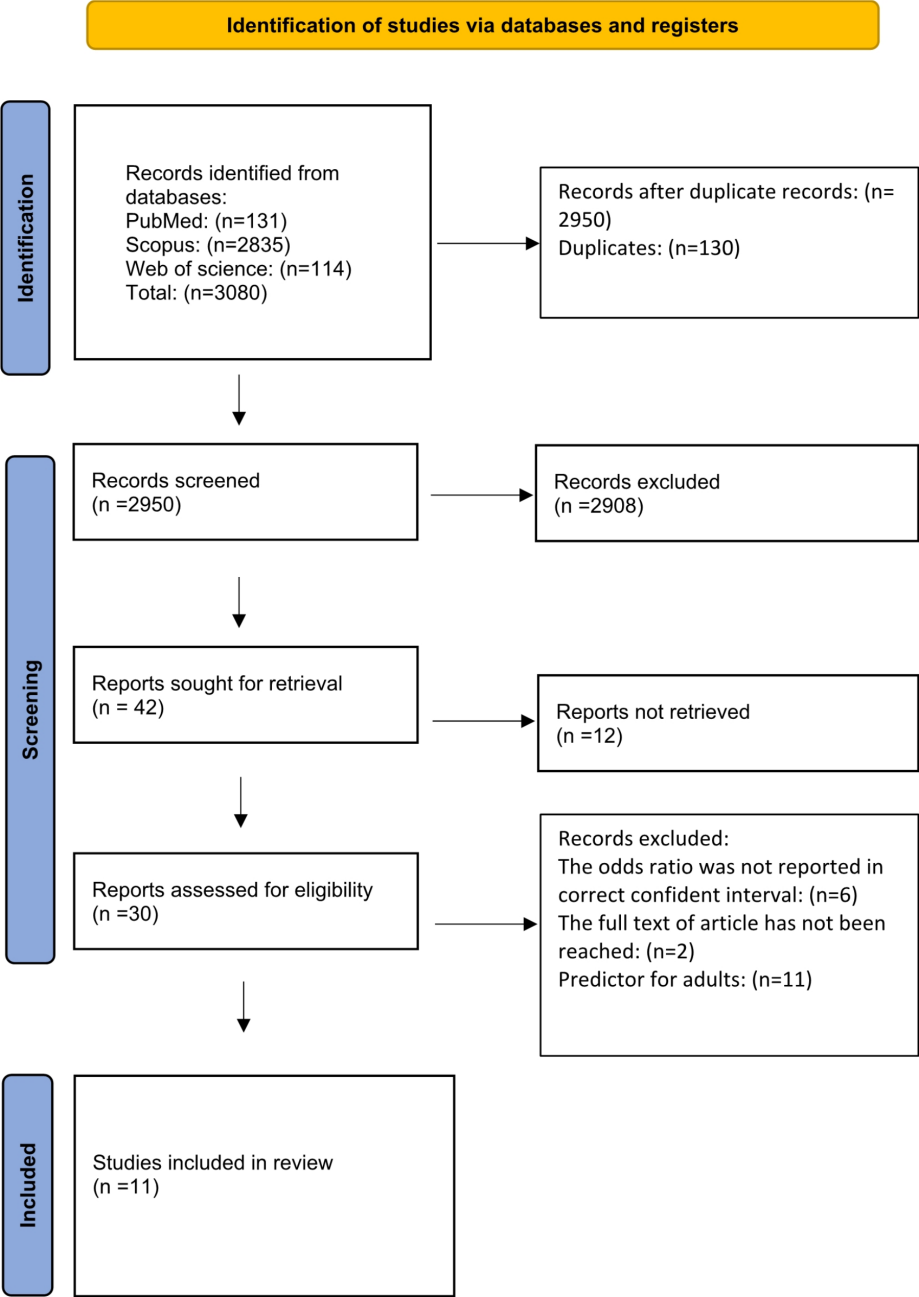
PRISMA diagram of the selection process

A comprehensive search of the literature was conducted in MEDLINE (through PubMed), Scopus, and Web of Science (ISI) up to November 2023 without any linguistic and time restrictions. The literature search was centered on observational studies that assessed the association between any type of SSB consumption and childhood asthma. The following keywords were used: (Sweetened beverage OR fruit drinks OR soda OR fruit juice OR high fructose corn syrup OR excess free fructose OR soft drinks) AND (asthma) (Table 1 in supplement). In addition, the reference list of relevant publications in other sources and gray literature manually was examined to prevent missing any eligible papers. All relevant publications were collected in the Endnote program and after removing duplicate studies as well as examining title and abstracts, full texts were examined to reach eligible articles (Fig. 1). This study was registered in PROSPERO with the code CRD42023483648.

**Table 1:** Summary characteristics of included studies

Cross sectional studies										
Author, year	country	sex	Sample size	exposure	Exposure assessment	Outcome assessment	Comparison (Highest vs. Lowest)	Effect size	Adjustment variables	Quality score
**Scheffers FR**,** 2022 [15]**	Netherland	Both	3046	SSB	FFQ	Doctor’s diagnosis- wheezing and asthma medication in the last 12 months.	> 14 gl/wk vs. 0-<7 gl/wk	1.18 (0.71–1.96)	Age-sex-educational level-parental history of allergy-breast feeding-physical activity-smoking-alcohol-vegetable-fruit-BMI	8
**Luyu Xie**,**2022 [24]**	US	Both	9938	SSB/ Fruit drink	24-h recall	Self-reported	Heavy consumption(> 500 kcal/d) vs. Never	SSB: 2.51 (1.55–4.08) Fruit drink: 1.89 (1.23–2.89)	Age-sex-race-family income-BMI	9
**Yueh-Ying Han**,**2019 [22]**	US	Both	24,612	Soda, pop	FFQ	Doctor’s diagnosis	> 7 times/ week vs. Never	1.25 (1.09–1.44)	Age-sex-race-BMI-average hour of sleep-fruit/vegetable intake-smoking-illegal drugs	9
**Lakiea S. Wright**,** 2018 [23]**	US	Both	1068	SSB/ TEFF	FFQ	Doctor’s diagnosis	> 5 times/ week vs. Never	SSB: 1.2 (0.74–1.94) TEFF: 1.53 (0.95–2.47)	Age-sex-race-maternal education-smoking during pregnancy-pregnancy BMI-income	7
**Melo B**,** 2018 [28]**	Brazil	Both	109,104	Soft drink	FFQ	Self-reported	> 5days/ week vs. 0-2days/ week	1.13 (1.05–1.22)	Sex-age-maternal education-smoker parents-smoke/alcohol consumption in past 30 days-school type-region-fruit/veg intake	9
**Luanne Robalo DeChristopher**, **2016 [14]**	US	Both	1961	TEFF/ Non-diet soft drink	FFQ	Self-reported	≥ 5 times/week vs. ≤1 time/month	TEFF: 5.29 (1.49–18.72) Non-diet soft drink: 1 (0.43–2.32)	Age-sex-race-BMI-total energy intake	7
**Danielle Saadeh**,**2015 [16]**	France	Both	7432	Fruit juice/ Soft drink	FFQ	Parental questionnaires- Skin prick testing	≥ 3 times/week vs. Never/occasionally	Fruit juice: 0.73 (0.56–0.97) Soft drink: 1.03 (0.77–1.37)	Sex-place of residence-parental atopic disease-sibling-maternal education-ethnic-breast feeding-smoke-obesity-day care center	8
**NE Berentzen**, **2015 [26]**	Netherlands	Both	2406	Sugar-added drink/ Energy drink/ Sport drink/ 100%fruit juice	24-h recall	Doctor’s diagnosis – prescription of inhaled corticosteroid- parental reports on wheezing	High (> 10gl/wk) vs. Low (< 4gl/wk)	Sugar-added drink: 1.41 (0.97–2.06) Energy drink: 1.36 (0.75–2.46) Sport drink: 1.14 (0.79–1.64) 100% fruit juice: 2.09 (1.21–3.6)	Age-sex-breast feeding-maternal education-parental allergy-smoking-fruit-vegetable-BMI	7
**HSIN-JEN TSAI**,**2007 [27]**	Taiwan	Both	2290	SSB	FFQ	Doctor’s diagnosis	Every day vs. Never	1.07 (1.03–1.12)	Sex-residential districts-allergy	8
**Cohort studies**										
**Luanne R. DeChristopher**, **2020 [12]**	US	Both	2097	Fruit drink/ soda/sports/ TEFF/ SSB	FFQ	Doctor’s diagnosis	> 2 times/ day vs. <2.5/ week	Fruit drink/ soda/sports: 2.57 (1.38–4.79) TEFF: 3.24 (1.69–5.67) SSB: 3.33 (1.92–5.75)	Sex-age-overweight-race-fruit/vegetable intake-expose to smoke-fast food-maternal education	7
**Margaret McCallister**,**2018 [25]**	USA	Both	164	SSB	FFQ	Doctor’s diagnosis- maternal report	> 5 times/week vs. 0–1 time/ month	1.13 (0.34–3.79)	Not adjusted	8

Abbreviation: SSB, sugar-sweetened beverages; TEFF, total excess free fructose; FFQ, food frequency questionnaire; gl/wk, glass per week; kcal/d, kilocalorie per day; BMI, body mass index

### Inclusion and exclusion criteria

Eligible articles and grey literature assessed the association between SSBs, including sports drinks, energy drinks, sugar-added drinks, soft drinks, fruit drinks, soda and pop, and other beverages containing high fructose, and also study which reported total excess free fructose (this outcome is referred to intake frequency of any combination of high fructose corn syrup sweetened soft drinks/sports/fruit drinks and 100% juice were summed to calculate the EFF according to the studies definition [14, 18, 19]) and asthma were included with these criteria: all types of observational studies design (cross-sectional, cohort, case-control studies), children population and adolescents lower than 18 years of age reported odds ratio (OR), hazard ratio (HR) or relative risk (RR) with 95% confidence interval (95% CI).

The exclusion criteria were: adult population, animal studies, any types of clinical trials studies, not English language, population with an acute condition like cancer, review articles, editorial, commentary or unpublished studies or abstract and statistical analysis reported correlation coefficients except estimated risk.

### Study selection

The study’s screening process involved independent evaluation by two reviewers, consistent with previous reporting. Initially, a title-abstract screening was performed by both reviewers, followed by a thorough review of the remaining titles deemed likely for inclusion. This meticulous approach ensured comprehensive scrutiny, with two reviewers independently conducting full-text screening for every potentially relevant paper identified.

### Data extraction

For the final evaluation of studies, we extracted the following data: First author, research publication year, study design (cross-sectional or cohort studies), participants’ age and sex, country of origin, sample size, exposure (highest vs. lowest), and outcome assessing method, effect size type and adjustment for different confounding factors. Data extraction was conducted independently by two reviewers.

### Risk of bias assessment

We evaluated the quality of articles by using the Newcastle-Ottawa Scale [17]. Based on three main domains of this checklist, including selection, comparability, and ascertainment of either exposure or outcome, each article can achieve a maximum of nine scores. We considered the score of 7–9 as high quality, 4–6 as moderate, and 1–3 as poor quality (Table 2 in supplement).

**Table 2:** Stratified analysis of the association between SSBs and asthma in children

	Number of effect size	Odds ratio(95%CI)	*P* value	*P*-heterogeneity	I^2^(%)
Overall	21	1.28 (1.15–1.42)	< 0.001	< 0.001	61.1%
**Age**					
$\:\ge\:$9	13	1.16 (1.07–1.25)	< 0.001	0.08	36.5%
< 9	5	1.13 (0.79–1.62)	0.49	0.61	0.0%
Both	3	2.25 (1.72–2.93)	< 0.001	0.59	0.0%
**Country**					
US	9	1.52 (1.23–1.88)	< 0.001	< 0.001	76.6%
Europe	10	1.18 (1.02–1.36)	0.02	0.90	0.0%
Asia	1	1.05 (0.99–1.11)	0.09	-	-
**Age adjustment**					
Yes	18	1.38 (1.21–1.57)	< 0.001	0.004	53%
No	3	1.05 (0.99–1.11)	0.10	0.65	0.0%
**BMI adjustment**					
Yes	12	1.36 (1.18–1.56)	< 0.001	0.22	21.9%
No	9	1.20 (1.04–1.37)	0.01	0.002	67.6%
**Energy adjustment**					
Yes	3	1.74 (0.99–3.06)	0.05	0.16	44.7%
No	18	1.23 (1.11–1.36)	< 0.001	0.002	55.5%
**Sample size**					
< 2000	4	1.13 (0.79–1.62)	0.49	0.61	0.0%
$\:\ge\:$2000	16	1.27 (1.15–1.45)	< 0.001	< 0.001	66.6%
**Design of study**					
Cross sectional	18	1.25 (1.13–1.38)	< 0.001	0.002	54.8%
Cohort	2	1.44 (0.33–6.22)	0.12	0.020	81.6%
**Dietary assessment**					
FFQ	12	1.14 (1.04–1.24)	0.002	0.08	37.9%
24-hour recall	9	1.54 (1.22–1.93)	< 0.001	0.04	50.6%
**Types of SSB**					
SSB	9	1.31 (1.08–1.58)	0.006	< 0.001	70.9%
Sugar drink	4	1.70 (1.29–2.24)	< 0.001	0.31	15.9%
Sport drink	4	1.12 (0.87–1.44)	0.38	0.79	0.0%
Soft drink	4	1.19 (0.96–1.47)	0.10	0.11	50.0%

Abbreviation: CI, confidence interval; BMI, body mass index; FFQ, food frequency questionnaire; SSB, sugar sweetened beverage;

The level of statistical significance was defined as p-value < 0.05

### Data analysis

To assess the link between SSBs, 100% fruit juice which is described as a natural fruit juice contenting high in fructose without any added sugar, total excess free fructose (tEFF), and risk of asthma in children. To convert HR to OR we used the following formula: (HR = OR / (1 - p + (p * OR)) for discerning risk ratio from odds ratio. Log risk estimate and standard errors (SEs) were calculated by using OR and 95% confidence interval (95%CI). Due to the high heterogeneity, the pooled OR was done in a random-effects model with DerSimonian and Laird method.

The I^2^ index, Cochran’s Q statistic, and associated P value were used to determine heterogeneity To examine heterogeneity, I^2^ > 50% is defined as heterogeneity status across studies. To identify the main sources of heterogeneity between studies, fixed model subgroup analysis was conducted based on the following criteria: continent (US, Europe, Asia), age ($\:\ge\:$9, < 9), age adjustment, BMI adjustment and energy adjustment, sample size ($\:\ge\:$2000, < 2000), design of the study (cross-sectional, cohort study) and type of SSBs (energy drinks, soft drinks, and sugar drinks, fruit drink). Egger’s test and funnel plot were used to assess the publication bias. All statistics were performed using STATA 17.0. level of statistical significance was defined as p-value < 0.05 [20].

## Results

During the initial phase of the search process, a total of 3080 studies were retrieved. Following the elimination of duplicate entries, the remaining articles underwent screening, resulting in the identification of 30 papers deemed eligible. However, the full text of two articles couldn’t be located, leaving 28 articles available for thorough examination. Subsequently, after excluding 11 articles about adult populations and an additional 6 articles with no reporting of odds ratio, a total of 11 articles were deemed suitable for inclusion in the final analysis (Fig. 1).

### Findings from the systematic review

#### Study characteristic

The main characteristics of the included articles are shown in Table 1. Nine articles were cross-sectional studies and 2 articles were cohort studies. All our included studies were published between 2007 and 2022. Six studies were performed in the USA [12, 14, 21–24], and other studies were done in Europe [15, 16, 25] and other countries [26, 27]. Both sexes were considered in all articles. The selected articles included 164 to 109, 104 participants (in total: 164,118). Two of the articles collected information about the number of consumed SSBs used the 24-hour recall [23, 25], while the rest used the FFQ (Food Frequency Questionnaire) [12, 14–16, 21, 22, 24, 26, 27]. Current asthma was defined by parental reports of physician-diagnosed asthma in children via questionnaire, based on the International Study on Asthma and Allergies in Childhood (ISAAC) [28], and also reports of wheezing or asthma medication in the last 12 months [15, 25]. Comparisons were obtained based on the highest vs. lowest intake of SSBs, tEFF, and fruit juice. The findings of 9 studies were adjusted for sex and age [12, 14–16, 21, 22, 25, 27], as well as BMI was the other adjustment factor in 6 studies [14, 15, 21–23, 25]. Most of the articles were adjusted for maternal factors like smoking [12, 15, 16, 21, 22, 25, 27], allergy [15, 25, 26], and maternal education [12, 15, 16, 22, 25, 27]. Dietary factors like fruit and vegetable intake [12, 15, 21, 25, 27], or fast food intake were considered too [12].

### Quality assessments

As we mentioned, the Newcastle-Ottawa Scale was used to evaluate the quality of articles [29]. According to the scores obtained by each paper, which were all above 7, all our articles are considered to be high quality.

### Findings from meta-analysis

**Fig. 2:**
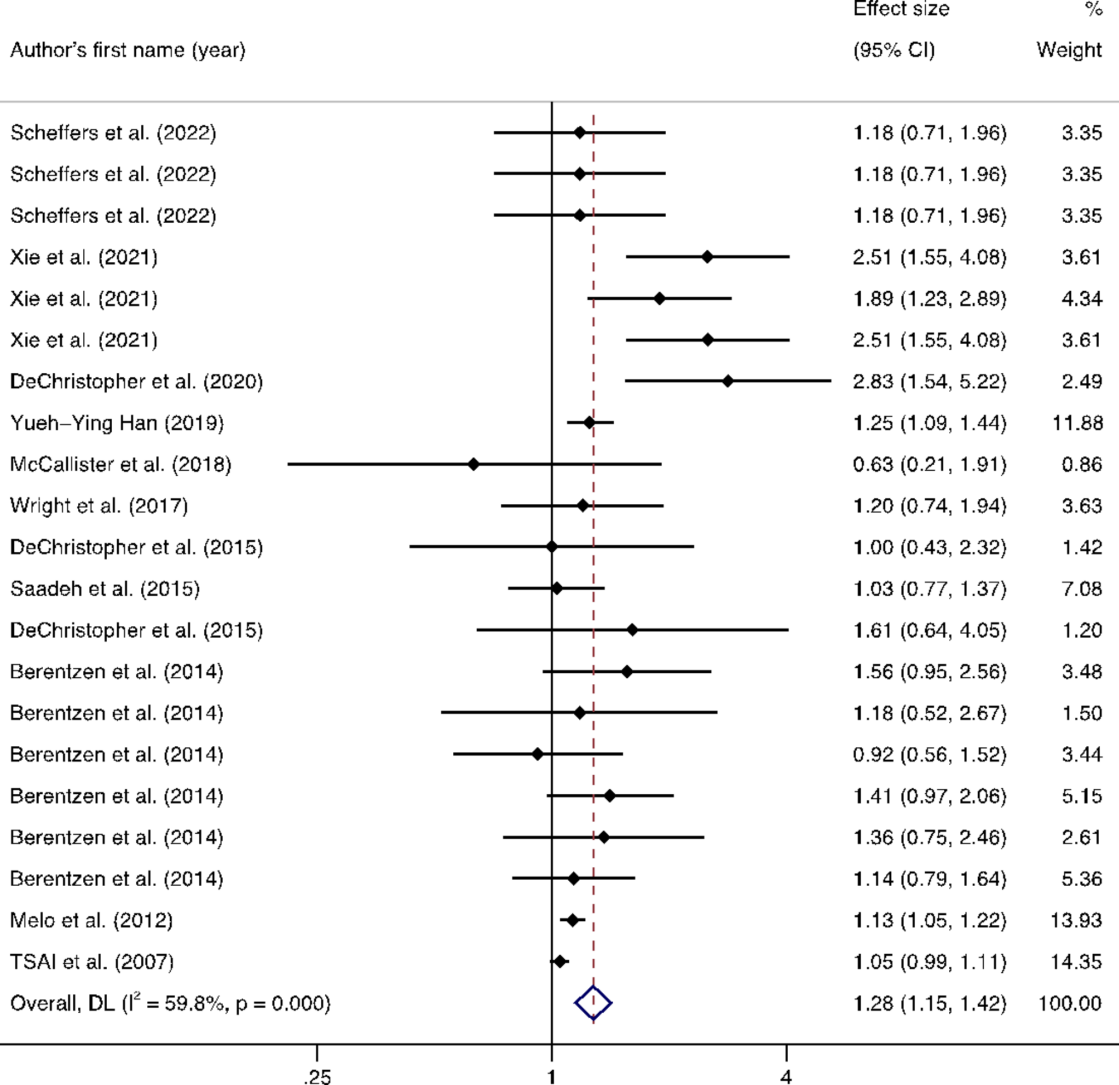
Estimated odds of sugar-sweetened beverages and asthma

**Sugar-sweetened beverages and asthma**: Twenty-one effect sizes with 39,857 individuals were included in the final analysis. It was shown that children who had higher consumption of SSBs had a 28% higher risk of asthma than children who had low or no (OR: 1.28; 95% CI: 1.15–1.42; P_value_ < 0.001) (Fig. 2). A moderate level of heterogeneity was found between studies (I^2^ = 59.8%; *P* < 0.001). We conducted subgroup analysis based on age, sample size, method of exposure assessment, adjusted by age, total energy intake and BMI, location of the study, design of the study, and different types of exposure (Table 2). After subgrouping, heterogeneity between SSB consumption and asthma remains significant in studies conducted in the US, studies with more than 2000 population, studies adjusted for age, and cohort studies. The overall effect size of the association between SSBs and risk of asthma did not depend on a single study. The funnel plot was significant for publication bias (Egger test intercept; *P* = 0.01) (Fig. 1 in supplement).

**Fig. 3:**
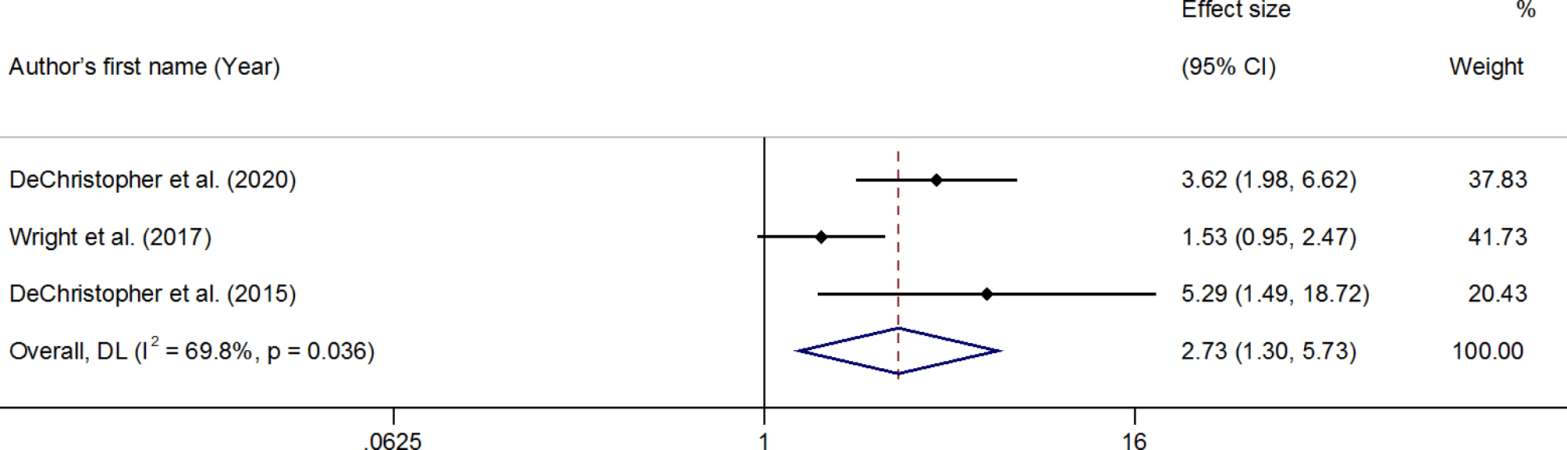
Estimated odds of total excess free fructose and asthma

**Total excess free fructose and asthma**: Three effect sizes with 5,126 individuals showed an association between consumption of tEFF and asthma in children. It was reported that children who intake a high amount of tEFF had a 2.7 times higher risk of asthma than children who had low or no consumption (OR: 2.73; 95% CI: 1.30–5.73; P_value_ =0.008) **(**Fig. 3). There was no evidence of publication bias (Egger test intercept; *P* = 0.46).

**Fig. 4:**
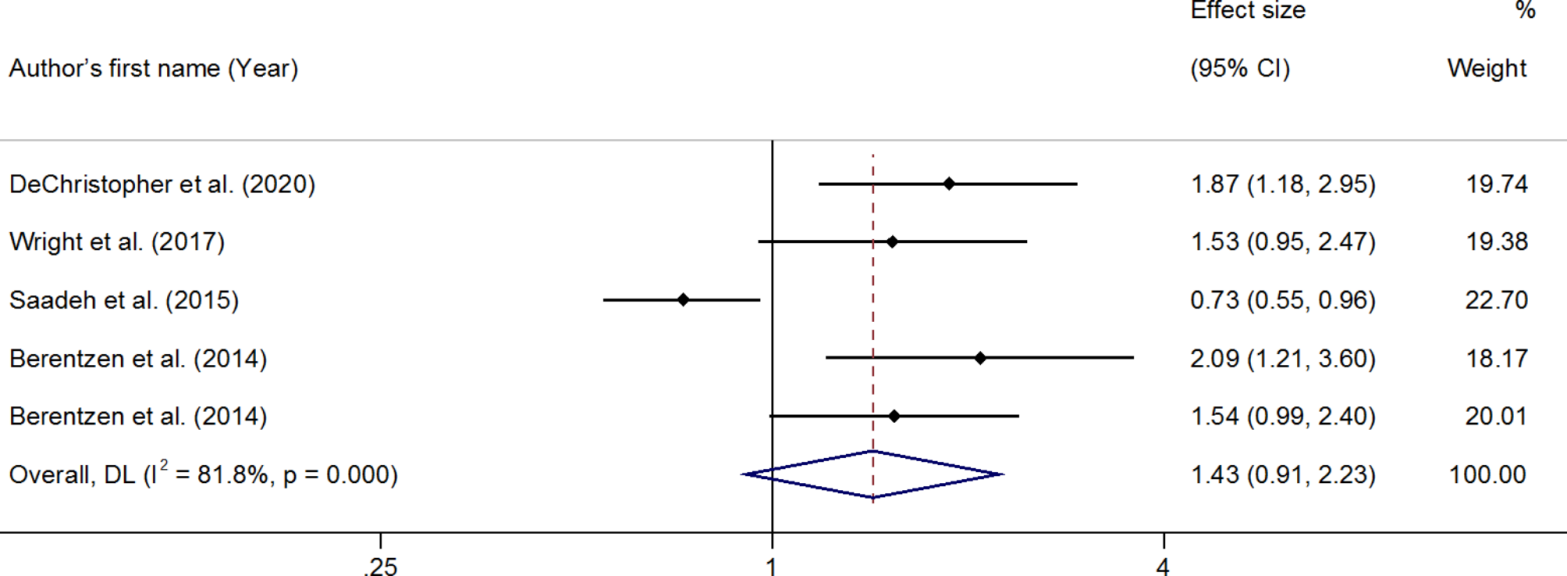
Estimated odds of 100% fruit juice and asthma in children and adolescents

**100% fruit juice and asthma**: Five effect sizes with 12,494 individuals indicated an association between fruit drinks and asthma which was included in the final analysis. It was shown that there is no statistically significant association between the consumption of fruit drinks and asthma in children (OR: 1.43; 95%CI: 0.91–2.23; P_value_ = 0.12) (Fig. 4). A high level of heterogeneity was found between studies. After subgroup analysis (Table 2), the association between fruit juice consumption and asthma changed to significant in studies that have been conducted in the US, studies adjusted for age and BMI, and studies with 24-hour recall assessment. The effect of the association between fruit drinks was decreased after removing one study (Table 3) [16]. The funnel plot was significant for publication bias (Egger test intercept; *P* = 0.003) (Fig. 2 in supplement).

**Table 3:** Stratified analysis of the association between Fruit juice and asthma in children

	Number of effect size	Odds ratio(95%CI)	*P* value	*P*-heterogeneity	I^2^(%)
**Overall**	5	1.43 (0.91–2.23)	0.14	< 0.001	81.8%
**Age**					
$\:\ge\:$9	4	1.34 (0.80–2.23)	0.26	0.001	83.1%
< 9	1	1.87 (1.18– 2.95)	0.007	-	0.0%
**Country**					
Europe	3	1.29 (0.66–2.50)	0.45	0.001	87.1%
US	2	1.69 (1.22–2.34)	0.002	0.95	0.0%
**Age adjustment**					
Yes	4	1.78 (1.36–2.35)	< 0.001	0.67	0.0%
No	1	1.03 (0.50–2.12)	0.93	0.009	85.5%
**BMI Adjustment**					
Yes	2	1.75 (1.22–2.51)	0.002	0.4	0.0%
No	2	1.25 (0.67–2.34)	0.47	< 0.001	87.1%
**Dietary assessment**					
FFQ	3	1.25 (0.66–2.35)	0.48	< 0.001	86.7%
24-hour recall	2	1.74 (0.91–2.23)	0.002	0.39	0.0%

Abbreviation: CI, confidence interval; BMI, body mass index; FFQ, food frequency questionnaire;

The level of statistical significance was defined as p-value < 0.05

## Discussion

Examining the result of our meta-analysis showed that, there was a direct significant association between the consumption of SSBs like sports drinks, TEFF, and fruit drinks and the risk of asthma prevalence among children. However, such an association was not seen between intake of fruit juice consumption and the odds of asthma. We observed an increasing trend in 100% fruit juice intake and asthma, although it didn’t reach significance. Heterogeneity across the studies could have influenced the results. As well as subgroup analysis showed increased odds for sugar-added drinks consumption and asthma prevalence but not for soft drinks.

Also, it is considerable from subgroup analysis that there is a significant association between SSB consumption and asthma in the US children population, especially those who are under 9 years [12, 14, 21–24].

Our study is the first systematic review and meta-analysis that assessed the association between the intake of SSBs and sugary drinks, which contain high amounts of fructose, and current asthma among children. Previous meta-analyses assessed the association between soft drink consumption and asthma, which combined both adult and child populations and didn’t specify the definition of the exact types of soft drinks [6]. In the present meta-analysis, we tried to include all types of sweetened beverages and fruit drinks that contain fructose, as a major contributor to asthma prevalence. Moreover, subgroup analysis was conducted to assess the association of each drink with asthma specifically.

Our result is consistent with previous articles that assessed the association between SSB consumption and childhood asthma. The study conducted by Han et al. showed that SSB consumption from drinking soda and pop sources significantly increases the odds of asthma in school children [21]. In addition, Wright et al. reported that higher intake of SSBs and total fructose in early childhood is associated with mid-childhood asthma development [22]. Moreover, the results of a cross-sectional study done by TSAI et al. indicated that among all food categories studied in this article, SSB consumption has the strongest association with asthma and six main respiratory symptoms in schoolchildren in Taiwan [26]. Also, the cohort study by McCallister et al. found that early consumption of SSBs leads to asthma prevalence after age 4 years, which can be due to the effect of drinking SSBs in increasing the risk of obesity. It indicates that intake of 500 calories per day by SSB consumption in children, increases the odds of asthma 2 times higher in comparison with non-SSB consumers [24]. A cross-sectional study conducted by Melo B et al. reported a positive significant association between intake of all ultra-processed foods including soft drinks and asthma prevalence in adolescents [27]. The results of this study were inconsistent with our results about soft drinks. However, the PIAMA cohort study found no association between SSB consumption and asthma prevalence in children 11–20 years old [15]. The controversial findings in this paper might be explained by the difference in study design and study population. It was a large cohort study with four measurement points from childhood until young adulthood and also assessed age-specific association, considering main lifestyle changes. Findings of subgroup analysis about SSBs show that drinking these beverages among US children under 9 years is considerable and if the consumption of SSBs in US children is not controlled, it leads to negative consequences [12, 22–24].

In line with our results, several studies found a positive association between TEFF and asthma. The study by DeChristopher et al. indicated that EFF intake from fruit drinks, non-diet soft drinks, and apple juice more than 5 five times a week increases the odds of asthma by five times in comparison to low or non-EFF consumption among 2–9 years old children. The correlation between EFF consumption and asthma in 10-16-year-olds was not significant. It seems that EFF tolerance increases with age [14].

Considering the relationship between fruit juice consumption and asthma, there is heterogeneity between the findings of different studies. The study by DeChristopher et al. showed that 100% fruit juice consumption, except orange juice, is associated with asthma. There was a significant association between 100% apple juice intake and asthma due to its high fructose-to-glucose ratio, which is associated with unabsorbed fructose [12]. In contrast, some studies support the protective effect of fruit juice on asthma development [30, 31]. A cross-sectional study conducted on French schoolchildren aged 9–11, showed an inverse association between 100% fruit juice consumption and asthma prevalence. This protective effect is due to the antioxidant and vitamin C content of fruit juice [15]. In line with our results, the PIAMA cohort study found no association between pure fruit juice consumption and asthma in 11-20-year-old children. However, it showed high intake of pure fruit juice (more than 7 times per week) increases the risk of asthma prevalence in 11-year-old children compared to low consumers [15]. The findings of our subgroup analysis showed, that drinking fruit juice among US children is significantly associated with asthma, which should be under more control to avoid its negative consequences.

Several mechanisms indicate the association between SSB consumption and asthma. One important mechanism is the ‘sugar hypothesis’ that shows an inflammatory pathway initiated by sugar presented in drinks, which increases the level of inflammatory markers like high-sensitivity C-reactive protein [32, 33]. On the other hand, high consumption of fructose from dietary drink sources activates inflammatory pathways in many tissues by promoting the expression of RAGE (receptor for advanced glycation end products) as a trigger for inflammatory reactions [34, 35]. When the percentage of fructose is higher than glucose, unabsorbed free fructose reacts with peptides in the lumen of the intestine and produces AGE (advanced glycation end products). Endothelial cell injury due to high consumption of fructose and activation of the Fructose-AGEs–RAGE axis eventually causes damage to lung tissue [36, 37]. In addition, SSBs can also cause overweight and obesity which are associated with the development of asthma, due to the reduction of the lung functional capacity by increasing hyper-responsiveness of airway smooth muscle following overweight and obesity [24, 38]. The last possible explanation is that the presence of sodium benzoate or potassium benzoate in sugar-sweetened beverages (SSB) and fruit drinks, but not in 100% fruit juice, may be responsible for wheezing and worsening asthma symptoms [39, 40].

### Strengths and limitations

Our systematic review and meta-analysis is the first report on the association of various types of dietary fructose with the risk of asthma. However, the study has some limitations. There is heterogeneity across study designs concerning population age and criteria for asthma. In addition, SSB and asthma assessment are mostly assessed by questionnaires, which are prone to recall and social desirability bias. Moreover, most of our included studies are cross-sectional, which means they cannot establish causality and tend to contribute to greater heterogeneity. Due to the novelty of the topic, the number of included studies is small, this may affect the meta-analysis results and lead to a low-power interpretation of the findings.

## Conclusion

According to the findings of the current meta-analysis of observational studies, SSBs, and tEFF consumption are associated with asthma risk in children and adolescents but there is no association between fruit drink consumption and asthma. More studies are needed to investigate whether the relationship between total excess free fructose and asthma is exclusively due to the presence of fructose or if there are other factors involved. In addition, well-designed larger cohort studies with a more limited age range are needed to be conducted.

## Electronic supplementary material

Below is the link to the electronic supplementary material.

Supplementary Material 1

## Data Availability

The [supplementary file] data used to support the findings of this study are included within the article.
