# Supplementary material for: A systematic review and meta-analysis of the association of all types of beverages high in fructose with asthma in children and adolescents
Source: BMC Nutr. 2024 Sep 18;10:123. doi: 10.1186/s40795-024-00930-1 (PMC11409540; doi:10.1186/s40795-024-00930-1)
Supplement: Supplementary file 1 — Supplementary Material 1 [file 40795_2024_930_MOESM1_ESM.pdf]

Supplementary file

A systematic review and Meta-analysis of the association of all type of beverages high in fructose with asthma in children and adolescents.

Table and figure content:

Excluded study explanation.

Table 1: Search strategy

Table 2: Risk of bias assessment with New Castle Ottawa.

Figure 1: Funnel plot of sugar-sweetened beverages (SSB).

Figure 2: Funnel plot of fruit juice.

Figure 3: Sensitivity analysis of SSB .

Figure 4: Sensitivity analysis of fruit juice.

Excluded studies:

The initial exclusion of articles was due to their lack of relevance to our topic of interest. Additionally, the articles were excluded if their exposure or outcomes were not relevant. In subsequent reviews, articles with different designs were eliminated [1-6]. Also, several articles, assessed the association between mother's intake of SSBs during pregnancy on childhood asthma were excluded [7-11]. Upon more detailed examination, articles that addressed our topic, but were conducted on adult population [12-22] or did not report the odds ratio [23] [24-28]were eliminated.

Supplementary table 1: Search strategy

Database	PubMed(n=131)	Scopus(n=2990)	Web of science(n=121)
Date	9th November	9th November	9th November
Strategy			
1	("soft drinks"[All Fields] OR "sugar sweetened beverage"[All Fields] OR "fruit juice"[All Fields] OR "high fructose corn syrup"[All Fields] OR "excess free fructose"[All Fields] OR "soda"[All Fields] OR "artificially sweetened beverage"[All Fields] OR "HFCS"[All Fields] AND ("excess"[All Fields] OR "excesses"[All Fields] OR "excessive"[All Fields] OR "excessively"[All Fields]) AND "free"[All Fields] AND ("fructose"[MeSH Terms] OR "fructose"[All Fields] OR "fructoses"[All Fields])) OR "ssb"[All Fields]) AND ("asthma"[MeSH Terms] OR "asthma"[All Fields] OR "asthmas"[All Fields] OR "asthma s"[All Fields])	ALL=("soft drinks") OR ALL=("sugar sweetened beverage") OR ALL=("fruit juice") OR ALL=("high fructose corn syrup") OR ALL=("excess free fructose") OR ALL=("artificially sweetened beverage") OR ALL=("HFCS") OR ALL=("fructose") OR ALL=("ssb") AND ALL=("asthma")	(TS=("soft drinks") OR TS=("sugar sweetened beverage") OR TS=("fruit juice") OR TS=("high fructose corn syrup") OR TS=("soda") OR TS=("artificially sweetened beverage") OR TS=("HFCS") OR (TS=("total") OR TS=("totaled") OR TS=("totaling") OR TS=("totaled")) OR (TS=("excess") OR TS=("excesses")) OR (TS=("free")) OR (TS=("fructose") OR TS=("fructoses")) OR (TS=("ssb")) AND (TS=("asthma") OR TS=("asthmas"))
2	("soft drinks"[All Fields] OR "sugar sweetened beverage"[All Fields] OR "fruit juice"[All Fields] OR "high fructose corn syrup"[All Fields] OR "excess free fructose"[All Fields] OR "soda"[All Fields] OR "artificially sweetened beverage"[All Fields] OR "HFCS"[All Fields] OR (("total"[All Fields] OR "totaled"[All Fields] OR "totaling"[All Fields] OR "totalled"[All Fields] OR "totalling"[All Fields] OR "totals"[All Fields]) AND ("excess"[All Fields] OR "excesses"[All Fields] OR "excessive"[All Fields] OR "excessively"[All Fields]) AND "free"[All Fields] AND("fructose"[MeSH Terms] OR "fructose"[All Fields] OR "fructoses"[All Fields])) OR "ssb"[All Fields]) AND ("asthma"[MeSH Terms] OR "asthma"[All Fields] OR "asthmas"[All Fields] OR "asthma s"[All Fields])	ALL((ALL (" soft drinks ") OR ALL (" sugar sweetened beverage ") OR ALL (" fruit juice ") OR ALL (" high fructose corn syrup ") OR ALL (" excess free fructose ") OR ALL (" soda ") OR ALL (" artificially sweetened beverage ") OR ALL (" HFCS ") OR ALL (" total ") OR ALL (" totaled ") OR ALL (" totaling ") OR ALL (" totaled ") OR ALL (" totals ") OR ALL (" excess ") OR ALL (" excesses ") OR ALL (" excessive ") OR ALL (" excessively ") OR ALL (" free ") OR ALL (" fructose ") OR ALL (" fructoses "))OR ALL (" ssb ")) AND ALL (" asthma ") OR ALL (" asthmas "))	(TS=("soft drinks") OR TS=("sugar sweetened beverage") OR TS=("fruit juice") OR TS=("fruit juice") OR TS=("high fructose corn syrup") OR TS=("soda") OR TS=("artificially sweetened beverage") OR TS=("HFCS") OR (TS=("total") OR TS=("totaled") OR TS=("totaling") OR TS=("totaled")) OR (TS=("excess") OR TS=("excesses") OR TS=("excessive") OR TS=("excessively")) OR (TS=("free")) OR (TS=("fructose") OR TS=("fructoses")) OR (TS=("ssb")) AND (TS=("asthma") OR TS=("asthmas"))

Supplementary table 2: quality assessment of included studies assessing the association between all type of beverages containing fructose and asthma in children.

Reference	Selection				comparability	Outcome	Score	
**Author, Year**	**Representativeness of the sample**	**Sample size**	**Non-respondent**	**Ascertainment of the exposure**	**Confounding factors are controlled**	**Assessment of the outcome**	**Statistical test**	**Total**
Floor R. Scheffers,2022	1	0	0	2	2	2	1	8
Luanne R. DeChristopher ,2020	1	0	0	2	2	2	1	8
Luyu Xie, 2021	1	1	1	2	2	1	1	9
NE-Berentzen ,2014	1	0	0	2	2	1	1	7
Yueh-Ying Han ,2019	1	1	1	2	2	2	1	9
Margaret McCallister ,2018	1	0	0	2	2	2	1	8
Lakiea S. Wright,2017	1	0	0	2	2	2	0	7
Luanne Robalo DeChristopher ,2015	1	0	0	2	2	1	1	7
Danielle Saadeh ,2015	1	0	0	2	2	2	1	8
Melo B, 2012	1	1	1	2	2	1	1	9
HSIN-JEN TSAI, 2007	0	0	1	2	2	2	1	8

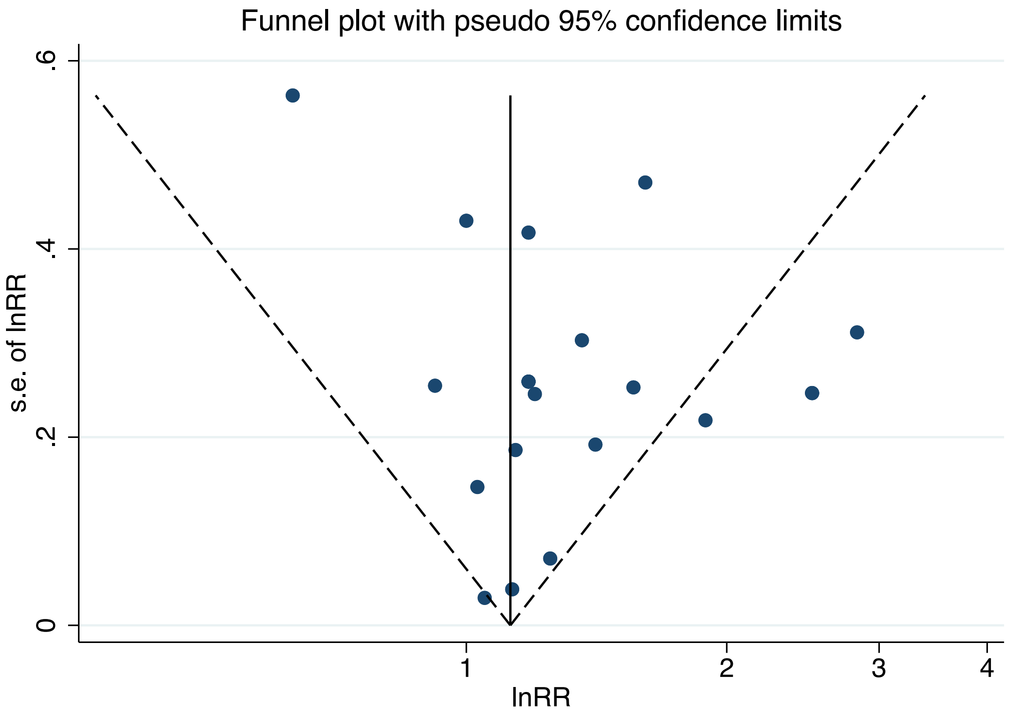

Supplementary figure 1: Funnel plot of SSB.

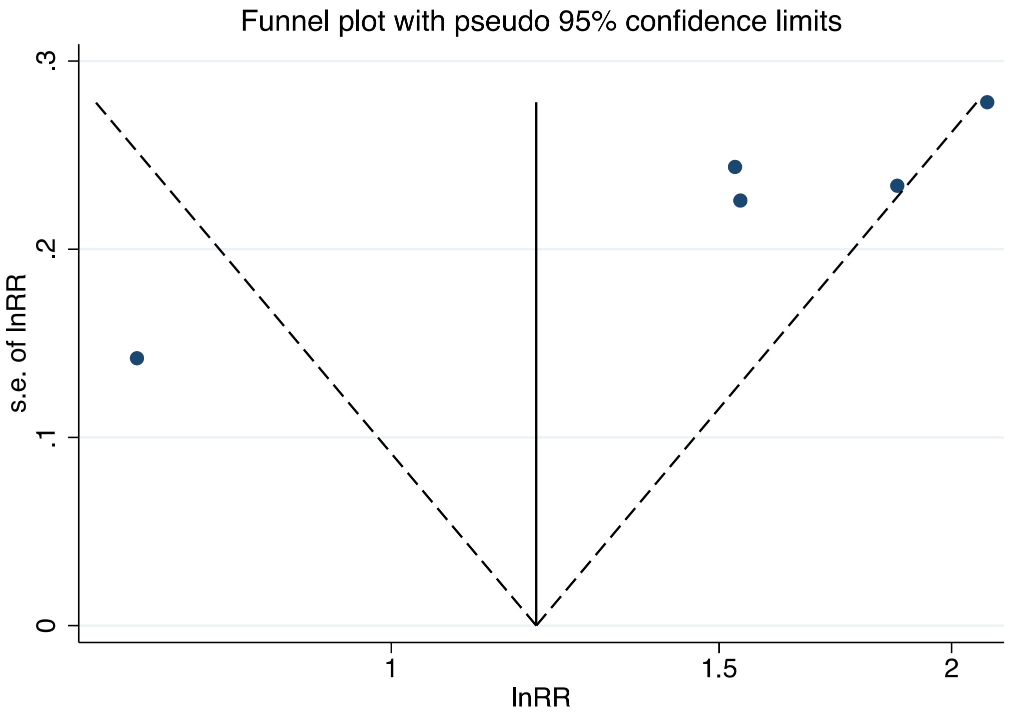

Supplementary figure 2: Funnel plot of fruit juice

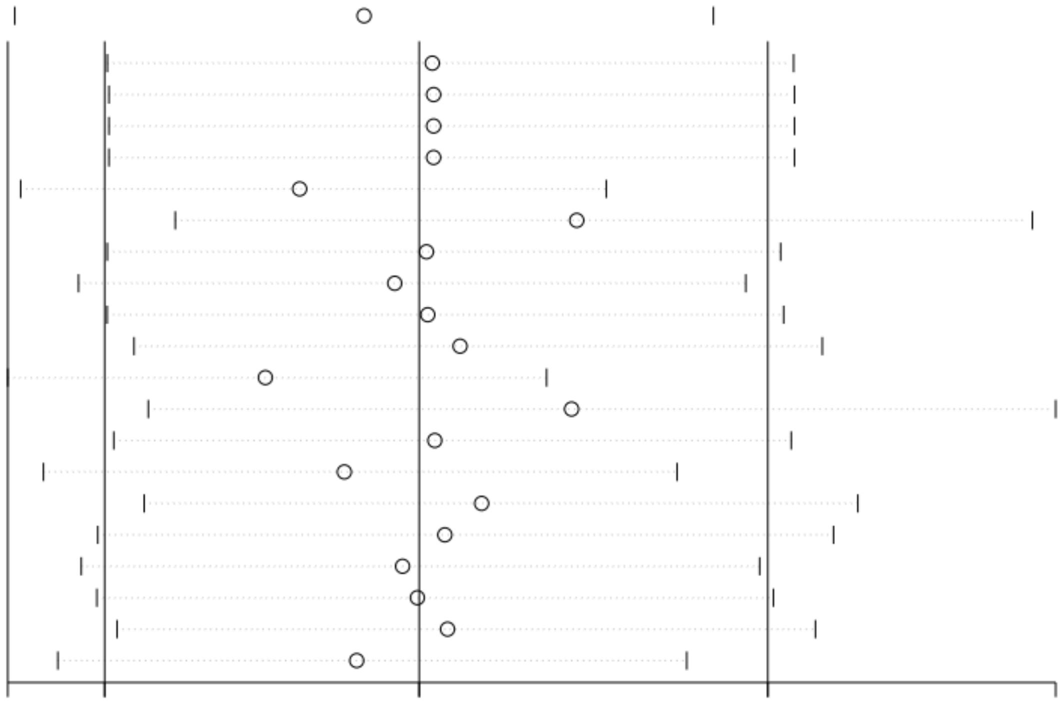

Supplementary figure 3: sensitivity analysis of SSB

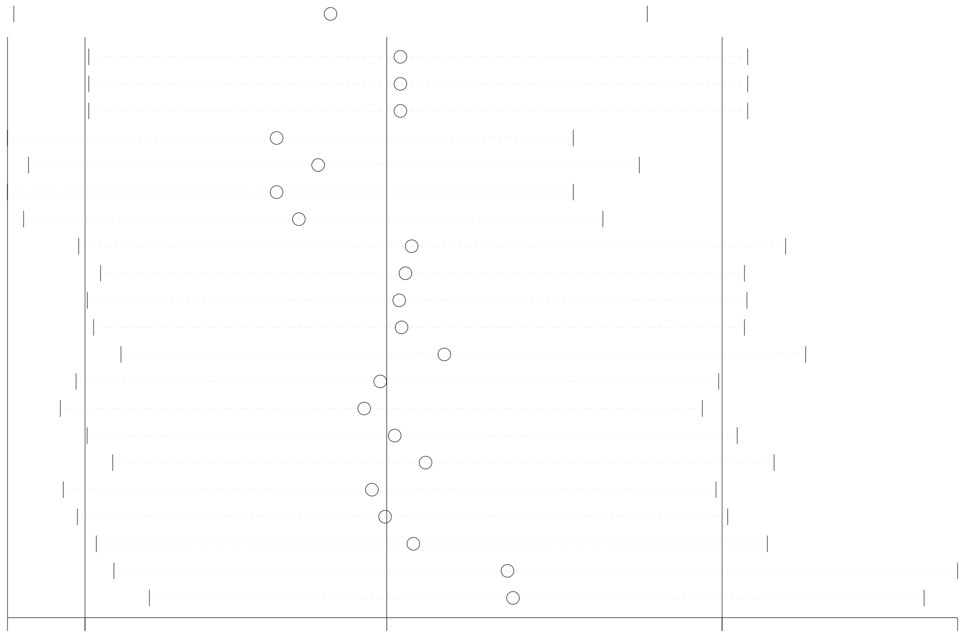

Supplementary figure 4: sensitivity analysis of fruit juice

1. Hosseini, B., et al., *Effects of fruit and vegetable consumption on risk of asthma, wheezing and immune responses: a systematic review and meta-analysis.* Nutrients, 2017. **9**(4): p. 341.
2. Khan, R., et al., *Consumption of Sugar-Sweetened Beverages and Their Health Impact on Children.* Discoveries, 2021. **4**: p. e17.
3. Hallit, S., et al., *Correlation of types of food and asthma diagnosis in childhood: a case–control study.* Journal of Asthma, 2018. **55**(9): p. 966-974.
4. Riordan, F., et al., *A systematic review of methods to assess intake of sugar-sweetened beverages among healthy European adults and children: a DEDIPAC (DEterminants of DIet and Physical Activity) study.* Public Health Nutrition, 2017. **20**(4): p. 578-597.
5. Al-Zalabani, A.H., et al., *Association between soft drinks consumption and asthma: a systematic review and meta-analysis.* BMJ open, 2019. **9**(10): p. e029046.
6. Toews, I., et al., *Association between intake of non-sugar sweeteners and health outcomes: systematic review and meta-analyses of randomised and non-randomised controlled trials and observational studies.* bmj, 2019. **364**.
7. Maslova, E., et al., *Consumption of artificially-sweetened soft drinks in pregnancy and risk of child asthma and allergic rhinitis.* PloS one, 2013. **8**(2): p. e57261.
8. Bédard, A., et al., *Maternal intake of sugar during pregnancy and childhood respiratory and atopic outcomes.* European Respiratory Journal, 2017. **50**(1).
9. Willers, S., et al., *Maternal food consumption during pregnancy and asthma, respiratory and atopic symptoms in 5-year-old children.* Thorax, 2007. **62**(9): p. 773-779.
10. Erkkola, M., et al., *Risk of asthma and allergic outcomes in the offspring in relation to maternal food consumption during pregnancy: a Finnish birth cohort study.* Pediatric allergy and immunology, 2012. **23**(2): p. 186-194.
11. Baïz, N., et al., *Maternal diet before and during pregnancy and risk of asthma and allergic rhinitis in children.* Allergy, Asthma & Clinical Immunology, 2019. **15**: p. 1-10.
12. Shi, Z., et al., *Soft drink consumption and multimorbidity among adults.* Clinical nutrition ESPEN, 2015. **10**(2): p. e71-e76.
13. Cisneros, R., et al., *Soda consumption and hospital admissions among Californian adults with asthma.* Journal of Asthma, 2017. **54**(4): p. 371-375.
14. DeChristopher, L.R. and K.L. Tucker, *Excess free fructose, high-fructose corn syrup and adult asthma: the Framingham Offspring Cohort.* British Journal of Nutrition, 2018. **119**(10): p. 1157-1167.
15. DeChristopher, L.R., J. Uribarri, and K.L. Tucker, *Intake of high fructose corn syrup sweetened soft drinks is associated with prevalent chronic bronchitis in US Adults, ages 20–55 y.* Nutrition journal, 2015. **14**: p. 1-8.
16. DeChristopher, L.R., J. Uribarri, and K.L. Tucker, *Intake of high fructose corn syrup sweetened soft drinks, fruit drinks and apple juice is associated with prevalent coronary heart disease, in US adults, ages 45–59 y.* BMC nutrition, 2017. **3**: p. 1-12.
17. Ibrahim, A.A., et al., *Association between soft drink consumption and asthma among Qatari adults.* Nutrients, 2019. **11**(3): p. 606.
18. Park, S., et al., *Association of sugar-sweetened beverage intake frequency and asthma among US adults, 2013.* Preventive medicine, 2016. **91**: p. 58-61.
19. Poongadan, M.N., N. Gupta, and R. Kumar, *Dietary pattern and asthma in India.* Advances in Respiratory Medicine, 2016. **84**(3): p. 160-167.
20. Wee, J.H., et al., *Analysis of the relationship between asthma and coffee/green tea/soda intake.* International Journal of Environmental Research and Public Health, 2020. **17**(20): p. 7471.

21. Troisi, R.J., et al., *A prospective study of diet and adult-onset asthma.* American journal of respiratory and critical care medicine, 1995. **151**(5): p. 1401-1408.
22. Barros, R., et al., *Dietary patterns and asthma prevalence, incidence and control.* Clinical & Experimental Allergy, 2015. **45**(11): p. 1673-1680.
23. Gilliland, F.D., et al., *Children's lung function and antioxidant vitamin, fruit, juice, and vegetable intake.* American journal of epidemiology, 2003. **158**(6): p. 576-584.
24. Padilha, L.L., et al., *Pathways in the association between sugar sweetened beverages and child asthma traits in the 2nd year of life: findings from the BRISA cohort.* Pediatric Allergy and Immunology, 2020. **31**(5): p. 480-488.
25. Emerson, S.R., et al., *The potential link between sugar-sweetened beverage consumption and post-exercise airway narrowing across puberty: a longitudinal cohort study.* Public Health Nutrition, 2016. **19**(13): p. 2435-2440.
26. Evans, E.W., et al., *Lung function, dietary intake, and weight status in children with persistent asthma from low-income, urban communities.* Nutrients, 2019. **11**(12): p. 2943.
27. Nascimento, J.X.P.T., et al., *The first 1000 days of life factors associated with "childhood asthma symptoms": Brisa cohort, Brazil.* Scientific reports, 2017. **7**(1): p. 1-12.
28. Thornley, S., et al., *Per capita sugar consumption is associated with severe childhood asthma: an ecological study of 53 countries.* Primary Care Respiratory Journal, 2011. **20**(1): p. 75-78.
